# Predicting Pre-planting Risk of Stagonospora nodorum blotch in Winter Wheat Using Machine Learning Models

**DOI:** 10.3389/fpls.2016.00390

**Published:** 2016-03-30

**Authors:** Lucky K. Mehra, Christina Cowger, Kevin Gross, Peter S. Ojiambo

**Affiliations:** ^1^Department of Plant Pathology, North Carolina State University, RaleighNC, USA; ^2^United States Department of Agriculture – Agricultural Research Service, RaleighNC, USA; ^3^Department of Statistics, North Carolina State University, RaleighNC, USA

**Keywords:** disease risk, machine learning, random forest, variable importance, Stagonospora nodorum blotch, wheat

## Abstract

Pre-planting factors have been associated with the late-season severity of Stagonospora nodorum blotch (SNB), caused by the fungal pathogen *Parastagonospora nodorum*, in winter wheat (*Triticum aestivum*). The relative importance of these factors in the risk of SNB has not been determined and this knowledge can facilitate disease management decisions prior to planting of the wheat crop. In this study, we examined the performance of multiple regression (MR) and three machine learning algorithms namely artificial neural networks, categorical and regression trees, and random forests (RF), in predicting the pre-planting risk of SNB in wheat. Pre-planting factors tested as potential predictor variables were cultivar resistance, latitude, longitude, previous crop, seeding rate, seed treatment, tillage type, and wheat residue. Disease severity assessed at the end of the growing season was used as the response variable. The models were developed using 431 disease cases (unique combinations of predictors) collected from 2012 to 2014 and these cases were randomly divided into training, validation, and test datasets. Models were evaluated based on the regression of observed against predicted severity values of SNB, sensitivity-specificity ROC analysis, and the Kappa statistic. A strong relationship was observed between late-season severity of SNB and specific pre-planting factors in which latitude, longitude, wheat residue, and cultivar resistance were the most important predictors. The MR model explained 33% of variability in the data, while machine learning models explained 47 to 79% of the total variability. Similarly, the MR model correctly classified 74% of the disease cases, while machine learning models correctly classified 81 to 83% of these cases. Results show that the RF algorithm, which explained 79% of the variability within the data, was the most accurate in predicting the risk of SNB, with an accuracy rate of 93%. The RF algorithm could allow early assessment of the risk of SNB, facilitating sound disease management decisions prior to planting of wheat.

## Introduction

Stagonospora nodorum blotch (SNB) of wheat (*Triticum aestivum*), caused by *Parastagonospora nodorum* (syn. *Septoria nodorum*), is a major disease of wheat worldwide (Solomon et al., 2006). The disease affects both the quantity and quality of yield, and losses up to 50% have been reported in susceptible cultivars (Eyal, 1981; Bhathal et al., 2003). In the United States, the disease is becoming more prevalent in part due to the increased adoption of minimum tillage (Shaner and Buechley, 1995), and losses of 30–50% have been reported during severe epidemics (Anonymous, 1995). Infected seed, ascospores from neighboring fields and wheat residue infected with *P. nodorum* serves can serve as sources of primary inoculum for infecting the wheat crop in the field (Holmes and Colhoun, 1975). Rain-splashed conidia are responsible for secondary infections during the season with temperatures between 15 and 25°C being conducive for disease development. Minimum tillage promotes the survival of *P. nodorum* in wheat residue left on the soil surface from the previous cropping season (Milus and Chalkley, 1997), a practice that ensures inoculum availability to initiate SNB epidemics at the start of the growing season. The cropping area under minimum tillage is increasing in wheat-growing regions of the United States (Horowitz et al., 2010). Minimum tillage can increase the likelihood of severe SNB epidemics, especially in rotations where wheat was planted the previous season, as is the case with wheat double-cropped with soybeans.

Stagonospora nodorum blotch is currently managed in wheat using a variety of methods that include crop rotation, tillage, planting moderately resistant cultivars, fungicide-treated seed, and foliar fungicides (Luke et al., 1983; Milus and Chalkley, 1997; Krupinsky et al., 2007). Pre-planting factors such as crop rotation and tillage have been shown to reduce the severity of SNB at the end of the season, but their effectiveness depends on their widespread adoption given that airborne ascospores from adjacent fields may lead to disease development even where there is no wheat residue on the soil surface (Cowger and Silva-Rojas, 2006). Foliar fungicides can be effective in controlling SNB, but in periods when wheat prices are low, the realized yield response may not be adequate to offset the cost of fungicide treatments (Weisz et al., 2011). Complete resistance in wheat to SNB is currently not available and cultivar resistance ranges from moderately resistant to highly susceptible. Cultivar resistance also interacts with wheat residue to influence SNB severity, with disease severity being higher on a highly susceptible than a moderately susceptible cultivar across a range of residue in the field (Mehra et al., 2015).

As for all plant diseases, an SNB epidemic is an outcome of an ecological process that involves the interaction between a population of host wheat plants and *P. nodorum*, a process that is influenced by the environment at different temporal and spatial scales (Madden et al., 2007). Variability in the susceptibility of the wheat plant population and pathogenicity of *P. nodorum* determine the extent of subsequent spread of SNB. Together with host plant resistance, the environment, defined broadly to include weather during the growing season and pre-planting factors that influence inoculum availability, determines the severity of SNB epidemics. Interaction among various elements of the pathosystem dictates that SNB epidemics will exhibit complicated behavior over different temporal and spatial scales (Madden et al., 2007). A fundamental goal in botanical epidemiology is to predict the risk of disease at various spatio-temporal scales (Madden, 2006). Information on the expected risk of a disease epidemic can aid growers in making better informed disease management decisions when seeking to reduce potential yield losses. Development of models to understand disease dynamics and predict the risk of disease outbreak to facilitate decision-making is an integral component of plant disease management (Scherm et al., 2006; Jeger and Xu, 2015).

Pre-planting factors such as crop rotation, type of tillage, cultivar resistance, and amount of wheat residue in the field can influence SNB risk during the growing season. Decisions pertaining to any of these factors singly or in combination can reduce the risk of SNB and its impact on yield at the end of the growing season. However, such a decision-making tool is currently not available for SNB. The choice to use a moderately resistant cultivar at a given location should be based on previous history of SNB at the location, whether wheat was planted the previous season and the type of residue management practiced in the field. Clearly, the use of moderately resistant cultivars in SNB management can be improved through a selective combination of host resistance with other pre-planting factors. For example, while the amount of wheat residue is related to disease severity, other factors such as cultivar susceptibility influence the magnitude of that relationship (Mehra et al., 2015). In addition, field location also appears to be an important pre-planting predictor of SNB. For example, in North Carolina, SNB tends to occur in the western (Piedmont region) and northeastern (Tidewater region) parts of the state (Weisz, 2013). Given that several pre-planting factors can potentially influence the risk of SNB, there is a need to develop a decision-making criterion that takes the effect of these factors, singly or in combination, into consideration. A pre-planting risk assessment model can provide critical information to guide SNB management decisions prior to planting of the wheat crop.

Predicting the risk of SNB by relating pre-planting factors to the severity of the disease can involve working with data that is complex and unbalanced. For example, the relationship between pre-planting factors (e.g., wheat residue) and SNB severity can be strongly non-linear and could involve high-order interactions with other factors (Mehra et al., 2015). When the interest lies in developing a model to predict a disease severity class, often the goal is to produce an accurate classifier for the disease class and to uncover the predictive structure of the problem. Traditionally, regression analysis has been the most popular modeling technique in predicting disease risk (De Wolf et al., 2003; Gent and Ocamb, 2009). In recent years, acccurate classifiers have been developed using machine learning methods, which are capable of synthesizing regression or classification functions based on available data (Gutierrez, 2015). Unlike traditional methods, machine learning methods can deal with complex and non-linear relationships between predictors and a response and are also able to process multifaceted and noisy data (Recknagel, 2001; Garzón et al., 2006).

Among machine learning methods, categorical and regression tree (CART) and artificial neural networks (ANN) methodologies have been used to predict the risk of plant diseases (De Wolf and Francl, 2000; Paul and Munkvold, 2004; Kim et al., 2014). The random forest (RF) technique (Breiman, 2001), which is an extension of CART, has been shown to have greater accuracy among machine learning methods (Svetnik et al., 2003; Garzón et al., 2006) and also provides a measure of the importance of each candidate predictor. RF has previously been used to predict invasion success of fungal pathogens in forests (Philibert et al., 2011). However, the algorithm has not been applied to asess the risk of disease development in agricultural systems. The overal goal of this study was to develop risk assessment models that can be used to guide management decisions for SNB before planting of the wheat crop. The specific objectives of the study were: (i) compare multiple regression (MR) and machine learning modeling techniques for their accuracy in predicting the risk of SNB using pre-planting factors and (ii) identify important pre-planting factors that influence the risk of SNB in winter wheat.

## Materials and Methods

### Field Sites and Data Collection

Field experiments were conducted at 12 sites in 11 counties in North Carolina during the 2011/12, 2012/13, and 2013/14 (hereafter referred to as 2012, 2013, and 2014, respectively) growing seasons (**Table 1**). Experimental sites were chosen to represent areas with different histories of SNB, varying cropping practices and a range of weather conditions. In each year, wheat was planted at each site in a conventionally prepared field, a no-tilled field, or both. Experimental plots across the study ranged between 1.0 to 1.5 m in width and 6.0 to 8.5 m in length. Crop production practices at each site followed standard recommendations for North Carolina (Weisz, 2013) but with no fungicide applications. Planting was earlier in the western than in the eastern part of the state, ranging from 25 September to 8 November in each year.

**Table 1:** Description of experimental sites and tillage methods used in a study conducted in North Carolina to identify pre-planting factors that influence the risk of Stagonospora nodorum blotch in winter wheat.

				Tillage method^a^		
Site	Field type^b^	County	Region	2012	2013	2014
Aurora	Grower	Beaufort	Tidewater	CT	–^c^	–
Caswell Farm	Research	Lenoir	South-Central	–	CT, NT	–
Cunningham Station	Research	Lenoir	South-Central	CT	CT, NT	CT, NT
Hertford	Grower	Perquimans	Tidewater	–	CT, NT	–
Lake Wheeler Road	Research	Wake	South-Central	CT	CT	–
Monroe	Grower	Union	Piedmont	–	NT	–
Piedmont Station	Research	Rowan	Piedmont	NT	CT	CT, NT
Tidewater Station	Research	Washington	Tidewater	NT	CT, NT	CT, NT
Tyner	Grower	Chowan	Tidewater	–	–	CT, NT
Rowland	Grower	Robeson	South-Central	CT	–	–
Upper Mt. Station	Research	Ashe	Piedmont	NT	CT, NT	–
Walkertown	Grower	Forsyth	Piedmont	–	CT, NT	CT, NT

*^a^CT = conventional tillage with complete burial of residue, and NT = no-tillage. ^b^Research fields were managed by personnel on the research station, while growers’ fields were managed by individual growers. In some instances, both conventional tillage and no-tillage experiments were conducted in separate fields at the same site. ^c^No experiment was conducted at the site in that year.*

In the 2012 season, the following five soft red winter wheat cultivars with resistance rating (RR) to *P. nodorum* ranging from 3 to 6 based on a scale of 1 (most resistant) to 9 (most susceptible) were used: Branson (RR = 6), Dyna-Gro Dominion (RR = 3), Dyna-Gro Shirley (RR = 4), SS8700 (RR = 3), and SY9978 (RR = 6). RRs were determined based on performance in the United States Department of Agriculture – Agricultural Research Service Septoria screening nursery in North Carolina (Anonymous, 2011). These cultivars had similar heading dates and generally possessed resistance to other foliar fungal wheat pathogens prevalent in the state. Cultivars were planted at each site in a randomized complete block design with six replicates.

In 2013 and 2014 seasons, two additional factors, seed treatment and seeding rate, were varied at each site. In these years, the experiment was laid out as a split–split–plot design with six replicates. Seed treatment was the main plot factor, seeding rate the sub-plot factor, and wheat cultivar the sub–sub–plot factor. Two levels of both the seed treatment factor, carboxin + thiram-treated or -untreated seed and the seeding rate, the standard rate (380 seeds m^-2^) versus a reduced rate (20% reduction of standard seeding rate) were evaluated. Seed was treated with imidacloprid insecticide to minimize the incidence of barley yellow dwarf virus. In 2013, four cultivars were used: Dyna-Gro 9012 (RR = 7), SS8641 (RR = 4), Dyna-Gro Shirley, and P26R20 (RR = 4). These four cultivars were also planted in 2014, except for SS8641 which was replaced by USG3438 (RR = 4). Cultivars tested in the first year of the study were replaced in subsequent years to generate a wide range of disease RRs across the entire study.

At each site, longitude, latitude, previous crop, and wheat residue cover on the ground were recorded. Latitude and longitude data were determined by locating the position of the field site on Google Maps (Google Inc., Santa Clara, CA, USA). The number 1 was assigned to fields where wheat was the previous crop, while 0 was assigned to fields where the previous crop was not wheat. The amount of wheat residue cover on the ground was determined using the line transect method (Wollenhaupt and Pingry, 1991). Briefly, a 100-feet long tape (with 1-foot interval markings) was stretched across each treatment block at a 45° diagonal. Residue cover for the treatment block was then determined by the number of times that a piece of residue intersected the tape at the 1-foot markings.

Disease severity was assessed visually on a whole-canopy basis by estimating the percentage of SNB severity in the plot (Mehra et al., 2015). Two to four assessments were made at most sites, while in a few cases, only one assessment was made due to either a shorter wheat season or low levels of disease. The response variable was maximum disease severity (MaxDS) averaged across replicates. In this study, MaxDS corresponded to disease severity at the last assessment date, recorded around the soft dough stage equivalent to Zadoks growth stage 85 (Zadoks et al., 1974). Values of MaxDS predicted from various modeling approaches were later categorized to generate a binary predicted response variable of low disease severity (<30%) and high disease severity (≥30%). The 30% severity cutoff on a whole-canopy basis corresponds to approximately 20% disease severity on the flag leaf (Mehra, unpublished results), which has been shown to result in yield loss in wheat (Bhathal et al., 2003). Thus, 30% disease severity is a useful threshold for risk assessment and making management decisions for SNB.

### Modeling Approach

Predictive modeling tries to find good rules for predicting the response variable based on the value of predictor variables in the dataset. In this study, MaxDS from a unique combination of predictor variables or disease cases (**Table 2**) was considered as the response variable. A total of 431 disease cases were obtained from the three years of the study across all experimental sites. Each modeling technique described below involved two independent steps. In the first step, the entire dataset was split randomly into training (70%), validation (20%), and test (10%) datasets using the procedure SURVEYSELECT in SAS (version 9.4, SAS Institute, Cary, NC, USA). This splitting was conducted 15 times (i.e., 15 randomizations of the data). Each time, a model was developed using the training dataset and optimized using the validation dataset, and the predictive ability of the model was tested on the test dataset. In the second step in the modeling process, the final model was developed using all the disease cases collected in the study.

**Table 2:** Independent variables tested for their usefulness in assessing pre-planting risk of Stagonospora nodorum blotch in winter wheat.

		Designation in modeling approach^a^	
Predictor variable	Type	CART, ANN, RF	Multiple regression (MR)
Cultivar resistance	Ordinal	1 to 9^b^	1 to 9^b^
Latitude	Continuous	Non-standardized	Standardized ^c^
Longitude	Continuous	Non-standardized	Standardized ^c^
Previous crop	Dichotomous	Wheat, no wheat	1 (wheat) or 0 (no wheat)
Seed rate	Dichotomous	Standard, reduced^d^	1 (standard), 0 (reduced)
Seed treatment	Dichotomous	Yes, no	1 (yes) or 0 (no)
Tillage type	Dichotomous	No-till, conventional	1 (yes), 0 (no)
Wheat residue	Continuous	Non-standardized	Non-standardized

*^a^CART = Classification and regression tree model, ANN = Neural networks model and RF = Random forest model. ^b^Cultivar resistance ranges from 1 (=most resistant) to 9 (=most susceptible). ^c^Latitude and longitude were standardized to have mean = 0 and standard deviation = 1, prior to fitting the MR model. ^d^Reduced seed rate is 80% of standard rate of about 380 seeds/m^2^.*

Two predictive modeling techniques, MR, and machine learning models, were applied in this study to predict the risk of SNB based on pre-planting variables. Within the machine learning paradigm, three predictive models were selected, ranging from the simple classification and regression trees to the more complex Breiman’s random forest algorithm.

During exploratory data analysis, previous crop, tillage type, and wheat residue were found to be highly correlated, while the three variables were not correlated with other pre-planting factors. Thus, the SAS PROC VARCLUS with option MAXEIGEN = 0.9 was used to eliminate two redundant predictors (Nelson, 2001). The VARCLUS variable reduction procedure identifies clusters of variables that are highly correlated among themselves but as uncorrelated as possible with variables in other clusters. Previous crop and tillage type were found to be redundant predictors and subsequently, only wheat residue and five other pre-planting factors (**Table 2**) were considered as independent variables in the ensuing modeling exercise.

### MR Model

Regression analysis is one of the most popular techniques for predictive modeling. A MR model with more than one predictor can be written as:

(1) $$y=\beta_{0}+\beta_{1}x_{1}+\beta_{2}x_{2}+\ldots +\beta_{m}x_{m+}\varepsilon ,$$

where *y* is the response variable (i.e., MaxDS), β_i_ is the regression coefficient, *x*_i_ is the *i*^th^ pre-planting predictor for *i* = 1,2,.., *m*, and 𝜀 is the random error term.

In the first step of the modeling process, the MR model was implemented using the SAS procedure GLMSELECT with the BACKWARD variable selection method and sub-options CHOOSE = validate, STOP = validate and MAXSTEP = 26 (Cerrito, 2006). The type and designation of predictor variables evaluated in the MR model is summarized in **Table 2**. To allow for direct comparison of model coefficients, latitude and longitude were standardized to have a mean of zero and a standard deviation of 1.0 prior to regression analysis (Schielzeth, 2010). Interactions between qualitative and quantitative predictors, and quadratic terms of quantitative predictors, were also included in the base model. The models obtained from the 15 randomizations of the training dataset were used to make predictions for the test dataset, and the prediction accuracy of the MR model was determined by linear regression of observed against predicted values of MaxDS.

In the second step of the modeling process, the final MR model was developed as described in the first step above using all the 431 disease cases. A significance level of 0.1 was used as the basis for variable retention. If a quadratic effect or an interaction was significant after variable selection, the main effects of variables comprising the quadratic effect or the interaction were included in the model to preserve the hierarchy. In the final step of the modeling approach, predicted values of MaxDS were assigned to the low- or high-disease severity class based on a disease severity threshold of 30%. The proportion of correctly classified cases, sensitivity (the proportion of true positives), and specificity (the proportion of true negatives) were then calculated.

### ANN Model

**FIGURE 1:**
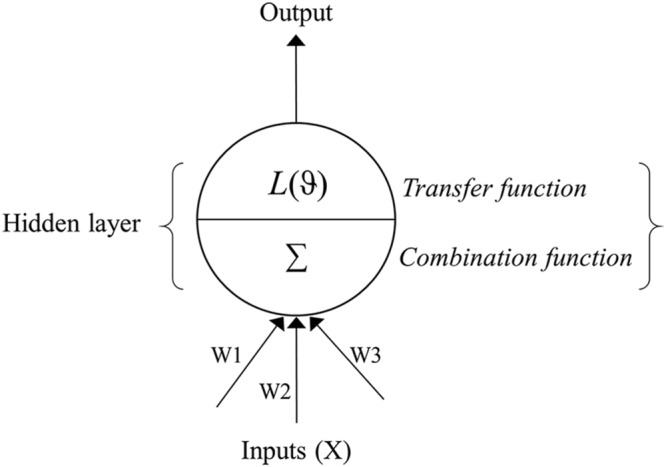
**Schematic flow of an artificial neural network (ANN) depicting the input, hidden and output layers.** The input layer contains predictors (*X_i_*), while the output layer contains the response variable. The hidden layer is composed of the combination (Σ) and transfer (*L*) functions and summarizes predictor variables and associated weights (W), applies the transfer function, and sends the result to the output layer. The weights (W1, W2, W3…etc.) link input and hidden layer of the neural network.

Artificial neural network models are analytical techniques that were originally developed by researchers attempting to mimic the neurophysiology of the human brain (Ripley, 1996). These models predict new cases after going through a learning process with existing data. An ANN is commonly divided into three or more layers: an input layer, a hidden layer(s), and an output layer (**Figure 1**). The input layer contains the input nodes (the input variables or predictors for the network), while the output layer contains the desired output of the system, and the hidden layer contains a series of nodes that are associated with transfer (or activation) functions. Each layer of the ANN is linked by weights that are determined through a learning algorithm.

A three-layer feed-forward network with back-propagation learning algorithm was used to predict the risk of SNB based on pre-planting predictors (Ripley, 1996; Francl, 2004). In the back-propagation algorithm, the weights of the network are trained by minimization of an error function (*E*) of the form:

(2) $$E\;=\;\frac{1}{2}\cdot [\Sigma {\left(t_{p}\;-\;y_{p}\right)}^{2}],$$

where *t* and *y* are the predicted and actual observed outputs, respectively, of the *p*^th^ training pattern (Rojas, 1996). A single-hidden-layer architecture was used, with the number of nodes in the hidden layer (*h*) serving as a tuning hyper-parameter of the whole model (Sarle, 2002). The logistic function of the form:

(3) $$L(\vartheta )\;=\;\frac{1}{1+e^{-\vartheta }},$$

was used as the activation function of the hidden layer that transfers the summed inputs to the output layer in which

(4) $$\vartheta \;=\;\sum_{i=1}^{n}w_{i}x_{i}\;+\;\theta_{i},$$

where w_i_ is the weight of the input value connecting to the hidden layer, x_i_ and 𝜃_i_ is the bias term (Venables and Ripley, 1999). The network approximation for the output (ŷ) is computed from

(5) $$y\;=\;\phi [\sum_{h}w_{h}\cdot L(\vartheta )(\sum_{i}w_{i}x_{i})],$$

where w_i_ is the weight of the hidden layer value connecting to the output layer and ϕ is the activation function of the output layer. In this study, ϕ was linear, resulting in linear units for the output of the ANN model. A logistic activation function of the hidden layer in combination with a linear function of the output layer generates good approximations of outputs in ANN (Venables and Ripley, 1999). The importance of predictors in the ANN model was determined using the Garson algorithm (Garson, 1991), as a recent study comparing methods for quantifying variable importance in ANN found this algorithm to be the most accurate (Fischer, 2015). The algorithm determines the relative importance of a variable by partitioning absolute values of hidden-output weights into components associated with each variable node and the importance of all variables sums to 100%. A weight decay value of 0.001 was used to avoid overfitting of the ANN by penalizing large weights that could increase the variance of output (Bishop, 1995). In implementing the ANN technique, the two independent modeling steps described above were conducted in the R statistical computing environment using the *caret* package. The tuneGrid function in *caret* was used to determine the number of nodes required in the hidden layer for optimal performance of the model. As recommended by Ripley (1996), the ANN model was implemented 100 times and the output from all the networks was averaged using the *avNNet* function within the *caret* package in R version 3.2.2 for Windows.

### CART Model

In the CART modeling technique, an empirical tree represents a segmentation of the data that is created by applying a series of simple rules. CART models generate rules that can be used for prediction through a repetitive process of splitting. Given a training dataset **L** with *N* cases, consisting of *m* predictors **X***_i_* (*i* = 1,..,*m*) as the input space **X** and the response variable *y*, the CART algorithm recursively partitions the input space to obtain a tree predictor (with y_η_′ as the predicted response for the sample X_η_):

(6) $$y^{\prime }\;=\;T_{L}(X_{\eta }).$$

Starting with the entire input space **X**, CART attempts to find a binary partition to increase the response purity in the subspaces formed by the partition. The partition is defined as a hyperplane perpendicular to one of the coordinate axes of **X**. The purity of the resulting subspaces depends on the homogeneity of the response classes. Several criteria are available to facilitate selection of the binary splits, depending on whether y_η_′ is a categorical or continuous response (Breiman et al., 1984). Binary partitioning is repeated in each new subspace until subspace response homogeneity is achieved. The maximal tree is usually overfitted and algorithms are used to constrain the overfitting by pruning the tree to its best generalization size. The prediction for a particular subspace is the majority vote (for classification if y_η_′ is categorical) or the average (for regression if y_η_′ is continuous) of training responses in that subspace.

In this study, y_η_′ was a continuous response variable and thus, a regression tree within the CART modeling approach was implemented using the ‘Decision Tree’ method in the ‘Partition’ modeling option in the JMP Pro statistical package (v11.2, SAS Institute, Cary, NC, USA). Given that the response variable *y* was a continuous response, binary partition was based on maximizing the *LogWorth* statistic:

(7) $$LogWorth=-log_{10}(P-value),$$

where *p*-value is the probability calculated from the sum of squares due to the differences in the means of the two groups formed from the partition (Su et al., 2009). A ‘validation’ column was provided to differentiate between the training, validation, and test datasets. The training and validation datasets were used to avoid overfitting the tree and to stop the splitting of tree nodes automatically when the coefficient of determination (*R*^2^) from the validation subset was better than the next ten splits (Breiman et al., 1984). A minimum split size of eight was specified in this study. The prediction formula was saved in the JMP data table, and used to predict MaxDS for the test subset. The prediction accuracy of the CART model was determined by simple linear regression of observed on predicted MaxDS values for the test dataset. Predicted values of MaxDS were then assigned to the low- or high-disease class, and the proportion of correctly classified cases, specificity, and sensitivity of the model were calculated as described above.

### RF Model

A RF is a collection of tree predictors, T_L_(X_η_;𝜃_k_), where *K* is the number of trees indexed by *k* = 1, … *K*; **X**_η_ is defined as above and has a vector length *p* associated with input vector **X;** and 𝜃_k_ are independent and identically distributed random vectors that indicate a training dataset **L**. The dataset **L** is assumed to be independently drawn from the joint distribution of (**X**, *Y*) and comprises *η* (*p* + 1)-tuples (**X**_1_,y1),…,(**X**_η_,y_η_). When the response is a continuous variable as in the present study, the final predictor **y**_η_ for a sample **X**_η_ is the average over all trees:

(8) $$y_{\eta }^{\prime }\;=\;\frac{1}{K}\sum_{k=1}^{K}T_{L}(X_{\eta };\theta_{k}).$$

As *K* →∞, the Law of Large Numbers ensures

(9) $$E_{x,y}{\left[Y-y_{\eta }^{\prime }(X)\right]}^{2}\;\to E_{x,y}[Y-E_{\theta }y_{\eta }{\left(X;\theta \right]}^{2},$$

in which the quantity on the right is the prediction error, and convergence in that equation ensures the lack of overfitting in RFs (Breiman, 2001).

To implement the RF model, the two independent modeling steps described above were conducted using the *randomForest* package in the R environment (Liaw and Wiener, 2002). The RF classifier requires the definition of two parameters for generating a prediction model: the number of classification trees desired (*K*), and the number of prediction variables (*m*) to select randomly at each node to make the tree grow. Here, *k* = {1, 2, …, *K*} trees were grown in the forest and the final predictor was the average across *K* trees. At each node within a tree, a given number of predictors was randomly chosen, and the best predictor was used to split the node. RF uses the Gini index to split a node and selects the split with the lowest impurity at each node (Breiman et al., 1984). The process was repeated across the subsequent nodes to grow the tree.

Each tree was developed using approximately two-thirds of cases as training dataset **L**, which was used to make a prediction for the remaining one-third of cases as the “out-of-bag” dataset. To control variance and overfitting, the number of predictors used at each node (*m* = 1 to 6) was evaluated using the function *tuneRF* of *randomForest* package in R and optimized using the “out-of-bag” error estimate (Liaw and Wiener, 2002). The *R*^2^ of the prediction on the out-of-bag dataset was taken as the prediction accuracy of the tree. A test dataset was also used in order to compare the RF model with the MR, ANN, and CART models. The RF algorithm also provides a measure of variable importance in the modeling, and the importance is derived from the contribution of each variable accumulated along all nodes and all trees where it is used (Breiman, 2001). The predicted values of MaxDS were assigned to the low- or high-disease class. The proportion of correctly classified cases, the specificity, and the sensitivity of the model were calculated as described above.

### Assessment of Model Performance

The Receiver Operating Characteristics (ROC) curve, i.e., a plot of 1-specificity vs. sensitivity rate, served to evaluate the performance of the models. Specifically, we estimated the area under the ROC curve (AUC), a threshold-independent index widely used in ecology. The ROC is based on the concept of class-dependent accuracy, which can be tabulated through a confusion matrix (McPherson et al., 2004). Points on the ROC are defined by the sensitivity and specificity indicators. The AUC ranges from 0.5 (random accuracy) to a maximum value of 1, which represents the most accurate model theoretically achievable. Two additional measures were calculated for each model: the coefficient of determination from regression of observed on predicted disease values, and the Kappa statistic (Monserud and Leemans, 1992). Kappa (κ) is a measure of agreement of model prediction beyond random chance and has a range of κ = 0 to 1. Values of κ: < 0.4 = low degree of similarity, κ: 0.40 to 0.55 = acceptable degree of similarity, while κ: 0.55 to 0.70 = good, 0.70 to 0.85 = very good, and >0.85 = excellent agreement beyond random chance (Monserud and Leemans, 1992).

## Results

A total of 431 unique disease cases were obtained from the field experiments in North Carolina, with 35, 236, and 160 cases recorded in 2012, 2013, and 2014, respectively. Based on our defined disease threshold, 297 (69%) were classified as low-disease cases, while 134 (31%) were classified as high-disease cases. The high-disease class contained 11, 42, and 19% of total cases in 2012, 2013, and 2014, respectively. Different modeling approaches were used to determine pre-planting factors that influenced the severity of SNB, and those factors were subsequently used to predict the risk of SNB. The results obtained for each predictive method and the accuracy of models developed are presented below.

### MR Model

The results of the MR analysis indicated that specific pre-planting predictor variables significantly contributed to the MR model (*F*-statistic = 51.55; *P* < 0.0001, *n* = 431). Cultivar resistance, longitude and wheat residue were the most important pre-planting factors identified by MR to influence the severity of SNB (**Table 3**). The quadratic effect of longitude also influenced the risk of SNB. Latitude, seed treatment with fungicide, and seeding rate were not significant (*P* > 0.05) predictors of MaxDS and were hence, not included in the final model.

**Table 3:** Results of MR analysis conducted to explain variation in maximum severity of Stagonospora nodorum blotch in winter wheat based on pre-planting variables using data collected in North Carolina from 2012 to 2014.

Variable	Estimate	Standard error	*t*-value	*P* > |*t*|
Intercept	0.20	2.157	0.09	0.9276
Cultivar resistance	2.24	0.401	5.60	0.0001
Longitude (LON)	1.99	0.569	3.49	0.0005
Wheat residue	0.05	0.017	3.21	0.0014
[LON]^2^	10.90	0.886	12.32	0.0001

**FIGURE 2:**
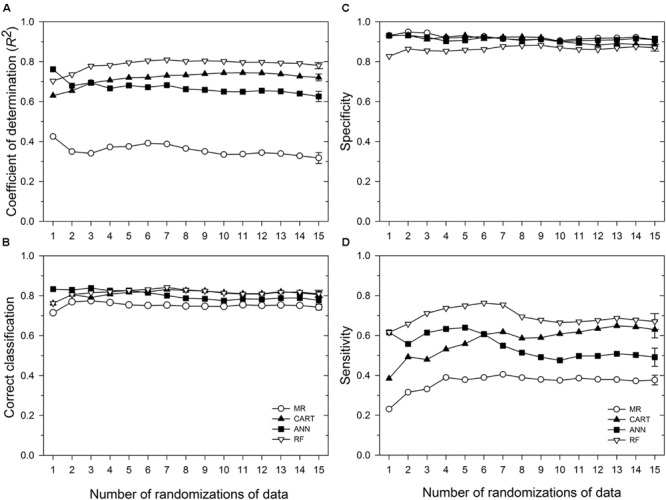
**Performance in predicting Stagonospora nodorum blotch (SNB) of wheat of a multiple regression (MR) model and three machine learning models: ANN, categorical and regression tree (CART), and random forest (RF).** Based on running averages of 15 randomizations of the test dataset which comprised 10% of 431 cases collected in the study. **(A)** coefficient of determination; **(B)** correct classification rate, i.e., proportion of cases correctly classified as low-severity (<30% disease severity) or high-severity (≥30% disease severity); **(C)** specificity, the proportion of cases correctly classified as low-disease; and **(D)** sensitivity, the proportion of cases correctly classified as high-disease. Symbols represent mean values for any given number of randomizations of the test dataset, while the vertical bars represent standard errors of the mean based on the total number of randomizations evaluated. Means whose standard error bars do not overlap are statistically different at α = 0.05.

Based on 15 randomizations of the test dataset, the proportion of variability (*R^2^*) of MaxDS in the test dataset explained by factors identified by MR was low with an average of 0.32 (**Figure 2A**). When MaxDS in the test dataset was classified as low- or high-disease, the average correct classification rate of the MR model was 0.74 (**Figure 2B**). The specificity of the model was very high with an average rate of 0.91 (**Figure 2C**), while average sensitivity was the lowest among four modeling approaches with an average rate of 0.38 (**Figure 2D**). The final MR model developed using all the 431 disease cases in the study explained 33% of the variation in MaxDS (**Table 3**). The final MR model had a correct classification rate of 0.74 (**Table 4**). The sensitivity of the final MR model was low with a value of 0.40, while the specificity was very high with a value of 0.90 (**Table 4**).

**Table 4:** Classification rates, sensitivity, specificity and prediction accuracy of final models developed using MR and machine learning techniques to predict the risk of Stagonospora nodorum blotch in winter wheat based on pre-planting variables using data collected in North Carolina from 2012 to 2014.

		Machine learning model^a^		
Test statistic	MR^a^	ANN	CART	RF
Coeff. of determination (*R*^2^)^b^	0.33	0.73	0.47	0.79
Correct classification^c^	0.74	0.83	0.83	0.81
Sensitivity^d^	0.40	0.60	0.55	0.69
Specificity^d^	0.90	0.93	0.95	0.86
AUC^e^	0.76	0.91	0.89	0.93
Kappa (SE)^f^	0.33 (0.049)	0.57 (0.044)	0.55 (0.044)	0.55 (0.043)

*^a^MR = Multiple regression model, ANN = Neural network model, CART = Categorical and regression tree model and RF = Random forest model. ^b^Coefficient of determination that indicates the amount of variation in MaxDS explained by the predictor variables from regression of observed on predicted disease values. ^c^Correct classification rate refers to the proportion of correctly classified cases within the dataset. ^d^Sensitivity is the proportion of cases correctly classified as high-disease (≥30% disease severity); Specificity is the proportion of cases correctly classified as low-disease (<30% disease severity). ^e^Area under the receiver operating curve, an estimator of the prediction accuracy of the model. ^f^Statistic for an estimator of the degree of agreement between observed values and model predictions beyond random chance; SE = standard error.*

### ANN Model

**FIGURE 3:**
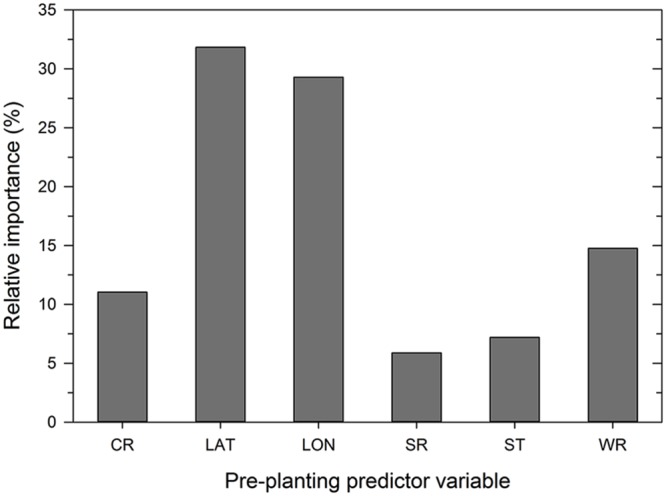
**Importance of pre-planting variables as identified by the neural network model.** Variable importance was determined using the ‘weights’ method of Garson (1991). CR = cultivar resistance rating (RR) for Stagonospora nodorum blotch, LAT = latitude, LON = longitude, SR = seeding rate, ST = see treatment, and WR = wheat residue on soil surface.

Analysis of the disease cases using the ANN methodology indicated that latitude, longitude, wheat residue, and cultivar resistance were the most important predictor variables (in decreasing order) that affected disease severity, with relative importance values ranging from >10 to 32% (**Figure 3**). Seeding rate and seed treatment were found to be of less importance (<10%) in the ANN (**Figure 3**). Increasing the number of nodes in the hidden layer reduced the root mean squared error (RMSE) and the final architecture of the ANN model was optimized at 12 nodes in the hidden layer based on lowestcross-validated RMSE.

Based on 15 randomized test datasets, the ANN model identified factors that explained a moderate proportion of the variability in MaxDS, with a mean of 0.63 (**Figure 2A**). MaxDS in the test dataset was correctly classified as low- or high-disease at an average rate of 0.78 (**Figure 2B**). The specificity of the ANN model was high with a mean rate of 0.91 (**Figure 2C**), while sensitivity of the model was low with an average rate of 0.49 (**Figure 2D**). When all the disease cases in the study were used for model development, the final ANN model accounted for 73% of the total variation in MaxDS (**Table 3**). Values of MaxDS predicted by the final ANN model and assigned to the low- or high-disease classes resulted in a correct classification rate of 0.83 (**Table 4**). The sensitivity of the final ANN model was moderate with a rate of 0.60, while the specificity was very high with a rate of 0.93 (**Table 4**).

### CART Model

The CART model selected based on the lowest Akaike’s Information Criterion had a total of 25 nodes. The tree was further pruned to seven terminal nodes without compromising the classification ability of the tree. The correct classification rates for the 7-node and 25-node trees were 0.83 and 0.85, respectively. The proportion of total variability in MaxDS explained by the CART model based on the test dataset was high with an average rate of 0.72 (**Figure 2A**). The correct classification rate of MaxDS in the test dataset was also high with a mean rate of 0.80 (**Figure 2B**). Based on the test dataset, the specificity of the CART model was high with an average rate of 0.88, while average sensitivity of the model was moderate with a mean rate of 0.63 (**Figure 2**).

**FIGURE 4:**
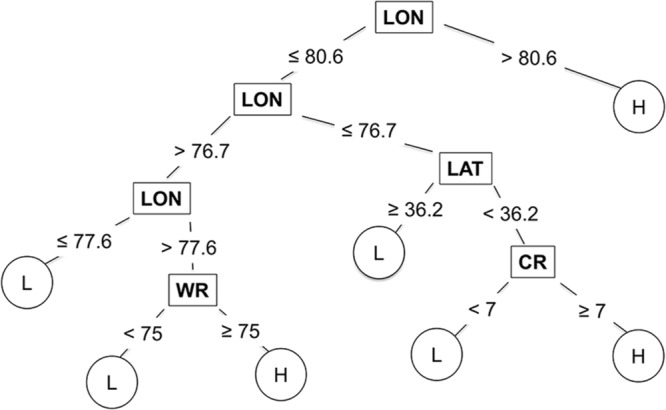
**Classification and regression tree used to estimate severity of Stagonospora nodorum blotch in winter wheat based on pre-planting predictor variables collected in North Carolina from 2012 to 2014.** Low (L) disease class = disease severity <30%, and high (H) class = disease severity ≥30%. The original tree had 25 nodes but was pruned to seven terminal nodes. Within the tree, predictor variables are shown in rectangles, while response variables are shown in circles. CR = cultivar RR for Stagonospora nodorum blotch, LAT = latitude, LON = longitude (degrees W), and WR = wheat residue on soil surface.

Longitude, latitude, cultivar resistance, and wheat residue were identified by the CART model as the most important predictor variables influencing the severity of SNB, and were used in the final CART model (**Figure 4**). Seeding rate and seed treatment were not identified as important factors affecting disease severity and were not used in the final CART model. The proportion of variation in MaxDS explained by the final CART model was 0.47 (**Table 4**). The average rate at which predicted values of MaxDS were correctly assigned to the low- or high-disease class based on the final CART model was high with a rate of 0.83. The final CART model had a moderate degree of sensitivity with a value of 0.55, while the specificity of the model was the highest with a value of 0.95 (**Table 4**).

### RF Model

**FIGURE 5:**
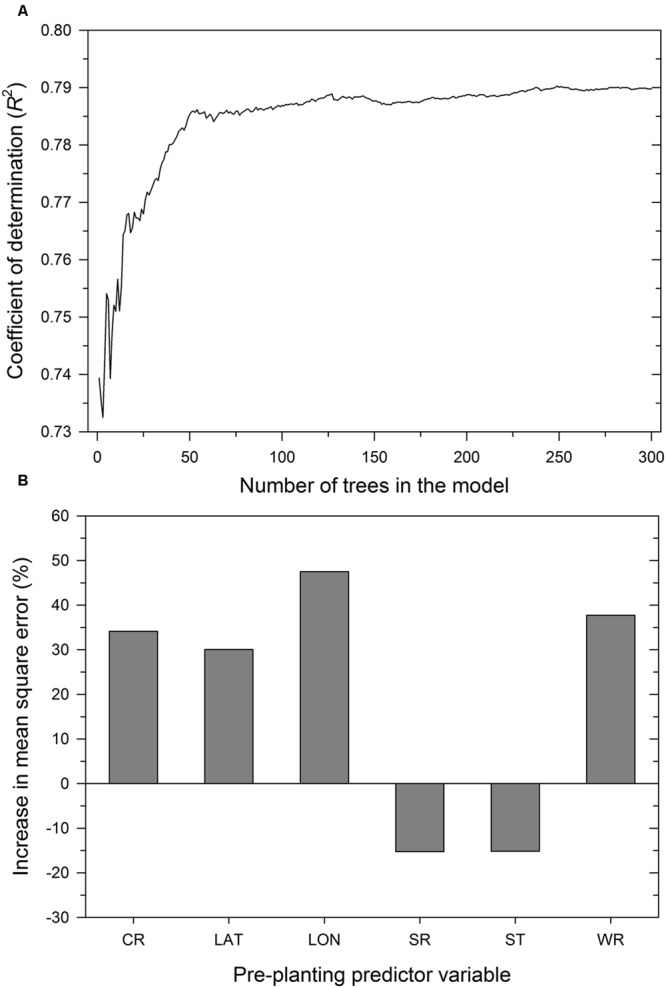
**Performance of the RF model depicting: **(A)** coefficient of determination as a function of the number of trees in the model and **(B)** importance of pre-planting predictor variables.** The importance of a predictor variable is determined by the percent increase in the mean square error after permuting a specific predictor variable. CR = cultivar resistance for Stagonospora nodorum blotch, LAT = latitude, LON = longitude, SR = seeding rate, ST = seed treatment, and WR = wheat residue on soil surface.

As expected, the number of trees in the RF model influenced the proportion of variability in MaxDS explained by the model. Increasing *K* from 1 to 30 trees increased *R*^2^ from 0.74 to 0.77 (**Figure 5**) and an additional increase in *K* from 31 to 100 resulted in a marginal increase in *R*^2^ to 0.79, The value of *R*^2^ stabilized with *K* ranging from 150 to 300 trees and the final RF model was obtained by aggregating 300 base models. The optimized number of variables used at each split in the final RF model was *m* = 3. In decreasing order of importance, the variables were longitude, wheat residue, cultivar resistance, and latitude (**Figure 5**). Like all other modeling methods, seeding rate and seed treatment were not identified by the RF model as having an important effect on disease severity.

Based on randomized test datasets, the proportion of variability in MaxDS explained by the RF model was significantly (*P* < 0.05) higher than that of all other models tested with an average of rate of 78% (**Figure 2A**). The average correct classification rate of MaxDS in the test dataset for the RF was 0.81 which was significantly (*P* < 0.05) higher than that of the MR and ANN models, but not significantly different from that of the CART model (**Figure 2B**). Based on the test dataset, the average specificity of the RF model was 0.87, which was significantly (*P* < 0.05) lower than that of other models tested except the CART model (**Figure 2C**). The sensitivity of the RF model was the highest among the four modeling techniques with a mean of 0.67, which was significantly (*P* < 0.05) higher than the sensitivity of other models except the CART model (**Figure 2D**). The final RF model explained the highest amount of variation in MaxDS with a value of 79% (**Table 4**). The rate at which predicted values of MaxDS were correctly assigned to the low- or high-disease classes in the final RF model was 0.81. The final RF model also had the highest sensitivity among all the models tested with a rate of 0.69 (**Table 4**).

### Model Performance

The accuracy of the models was assessed based on coefficients of determination of the final models, sensitivity-specificity ROC analysis, and the Kappa statistic. As indicated above, the final RF model had the highest coefficient of determination followed by the ANN and CART models, while the *R*^2^ value for the MR was the lowest (**Table 4**). The RF also had the highest area under the ROC curve (AUC = 0.93), followed by the ANN and CART models, while AUC value for the MR model was the lowest with an AUC of 0.81 (**Table 4**). The MR model had the lowest Kappa value (κ = 0.37), which indicates a low degree of agreement of MR model predictions beyond random chance. However, Kappa values for CART, ANN, and RF models were comparably higher with values ranging from κ = 0.55 to 0.57 (**Table 4**), which indicates an acceptable degree of agreement of the three machine learning model predictions beyond chance.

## Discussion

Pre-planting factors previously correlated with the late-season severity of SNB (e.g., Luke et al., 1983; Mehra et al., 2015) were used to develop risk assessment models that could be useful in making disease management decisions prior to planting of the wheat crop. Two analytical techniques, MR and machine learning, were used to develop models to predict MaxDS from eight pre-planting predictor variables using data collected across diverse ecological conditions, disease histories and cropping practices in North Carolina. Models developed in this study identified longitude, latitude, cultivar resistance and amount of wheat residue as significant predictors of SNB severity. Assessment of the accuracy of the models using the ROC curve analysis showed that the RF model was the most accurate classifier for assessing the risk of SNB. To our knowledge, this work represents the first use of RF to predict disease risk in plant-based systems and the first study to develop pre-planting risk assessment models for SNB in wheat.

In winter wheat, the role of field location, previous crop, cultivar resistance, amount of wheat residue on the soil surface, seed treatment, and tillage in the development of SNB is well documented (King et al., 1983; Luke et al., 1983; Leath et al., 1993; Stover et al., 1996; Milus and Chalkley, 1997; Solomon et al., 2006; Weisz, 2013). However, the relative importance of each of these factors to the severity and risk of SNB has never been determined. In addition, a clear understanding of the most important pre-planting factors that influence the risk of SNB was previously lacking. The three machine learning models developed in this study identified longitude, latitude, cultivar resistance, and wheat residue as significant predictors of the SNB severity. The MR model also identified all these factors, except latitude, as significant predictors of SNB. These results also validate previous reports on the effect of location, cultivar resistance and wheat residue on the severity SNB (e.g., Holmes and Colhoun, 1975; Luke et al., 1983; Weisz, 2013), and indicate that these predictors are useful in predicting the risk of SNB in winter wheat.

Stagonospora nodorum blotch is frequently problematic in the western and northeastern parts of North Carolina (Weisz, 2013). Thus, it is not surprising that longitude was an important predictor of SNB in North Carolina, with all the models identifying longitude as one of the two most important predictors, along with latitude. The effect of longitude on SNB severity can be seen directly in the MR model by the significant quadratic effect of standardized longitude which indicates that predicted disease severity is higher in the east and west and lower in the central parts of the state. This was especially evident under conventional tillage, where high levels of SNB were observed in western parts of the state (data not shown). The importance of longitude as a predictor can be explained by variation in environmental conditions that favor the development of SNB. In this study, rainfall amounts recorded in experimental sites in the eastern or western part of the state were 10 to 80% higher than in sites located in the central part of the state (Mehra et al., unpublished results). The level and frequency of precipitation are both known to favor the development of SNB in wheat (Verreet and Hoffmann, 1990). The median latitude was slightly higher in the high-disease class than in the low-disease class but the effects of latitude on SNB severity were highly dependent on the previous crop.

Previous crop, tillage and wheat residue were highly correlated and wheat residue was identified as the best predictor for the risk of SNB among these three pre-planting factors. The limited importance of previous crop and tillage could be explained by the fact that these two variables are an indirect measure of the survival of *P. nodorum* from one season to the next, a characteristic that is better reflected by wheat residue. Similar observations were also reported in a study that evaluated the importance of pre-planting factors for the risk of gray leaf spot of maize (Paul and Munkvold, 2004). In addition, even under conventional tillage with incomplete burial of residue, 10% of residue remains on the soil surface (Stubbs et al., 2004), which can result in an end-of-season disease severity similar to that in fields with 30% residue that can result from no-till fields (Mehra et al., 2015). The amount of residue in no-till fields also depends on the previous crop, with higher residue levels resulting when wheat is planted after wheat as compared to when another crop is planted between two wheat crops. The tillage effects on disease severity in the latter case would not be easily distinguishable from the effects of tillage with complete burial of residue since both practices would result in little or no residue to influence disease severity. None of the modeling approaches identified seed treatment or seeding rate as useful predictors of SNB risk. The seed used in the present study was certified and was free of *P. nodorum*, which explains why seed treatment was not an important factor. The reduced seeding rate used in this study may not have been enough lower to generate a significance difference in SNB compared to the normal seeding rate. Further reductions in seeding rates may result in high SNB compared to the normal rates, but growers are not likely to use such low seeding rates due to potential yield penalties.

Predicting the risk of disease plays an important role in the decision making and planning process for disease management in plant pathosystems. Historically, regression models have been widely used to predict epidemics of plant diseases (Rosso and Hansen, 2003; Uddin et al., 2003; Paul and Munkvold, 2005; Del Ponte et al., 2006; Olatinwo et al., 2008). In this study, while the MR model had a very high specificity and thus, was a very good predictor of low disease severity at the end of the season, the model explained less than 50% of total variability in the dataset. The MR model was also a poor predictor of the high-disease class as evidenced by its low sensitivity. The MR model was also the least accurate in predicting the risk of SNB as indicated by its lowest AUC. Highly accurate decision rules that combine high levels of sensitivity and specificity will be required for any predictive system to be useful in management of plant diseases (Gent et al., 2013). Regression analysis has the advantage of simplicity and produces a model equation with parameter estimates that can be directly related to scientific hypotheses and thus, has been the main choice for modeling disease risk in botanical epidemiology. However, other approaches such as ANNs and decision trees have been useful in developing predictive models in several scientific fields (Gutierrez, 2015). Application of these alternative methods has been limited but is slowly gaining interest in plant disease epidemiology.

Artificial neural networks have been used to model the risk of disease development in plant-pathosystems (Batchelor et al., 1997; De Wolf and Francl, 2000; Chakraborty et al., 2004). However, applications of ANN in these systems did not establish the relative importance of predictor variables. The ANN algorithm used in the present study allowed for estimation of the relative importance of each predictor variable in assessing the risk of SNB. The ANN model developed here performed much better than the MR model, with a good balance between model sensitivity and specificity. Often, prediction is more important than explanation in standard back-propagation ANN models, and model construction is not easily understood (Frasconi et al., 1993; Hastie et al., 2009), which has created a perception of a ‘black box’ that has limited the use of ANN models. The inability to easily calculate standardized coefficients for each independent variable and the difficulty in interpreting weights from the network analysis are also other weaknesses of ANN models (Frasconi et al., 1993; Ottenbacher et al., 2004). In this study, transparency was increased and the explanatory power of the ANN model was improved by determining the relative contribution and importance of each predictor variable to the prediction of SNB severity. The number of nodes in the hidden layer required in the optimized model was twelve, which was greater than the number of important predictors in the model. This suggests that the relationship between MaxDS and pre-planting variables is non-linear and hence will not be fitted well by the MR model without adding higher-order interactions and polynomial terms. In contrast, nodes in the hidden layer of the ANN model intrinsically captured the non-linearity between MaxDS and predictor variables.

The CART modeling technique has previously been applied to predict the risk of disease in plant- and forest-pathosystems (Rosso and Hansen, 2003; Paul and Munkvold, 2004; Fan et al., 2006; Kelly et al., 2007; Copes and Scherm, 2010; Kim et al., 2014). In this study, the CART model performed better than MR and was as accurate as the ANN model in predicting the risk of SNB. Simplicity of the modeling approach is one notable attribute of CART that allows for determination of variable importance at each node (De’ath and Fabricius, 2000). CART also generates an intuitive tree diagram that illustrates the relationship between the response and the predicted variables. The tree indicated that several combinations of predictor variables could result in the same disease-severity class, and that longitude, latitude, wheat residue and cultivar resistance strongly influenced MaxDS. High levels of SNB occurred west of longitude 80.6, which is the Piedmont and foothills of the Appalachian Mountains, while low and high severity occurred east of longitude 80.6 (the Coastal Plain and Tidewater regions) depending on the latitude, amount of wheat residue and cultivar resistance. For example, east of longitude 76.7 (in the Tidewater), low disease occurred north of latitude 36.2 (roughly, north of the Albemarle Sound), while high disease occurred south of latitude 36.2 when a highly susceptible cultivar was planted. The final CART model, pruned to seven terminal nodes, predicted SNB classes as well as the fully grown tree with 25 terminal nodes, and thus, it is likely to generalize well on an independent dataset (Breiman et al., 1984).

The RF algorithm, which has previously not been used to predict the risk of disease in plant-based agricultural systems, produced the most accurate model to predict the risk of SNB in winter wheat. Like the other models evaluated, RF identified location, wheat residue and cultivar resistance as the key predictors affecting the risk of SNB. The key advantages of RF include its non-parametric nature, high classification accuracy, and capability of determining variable importance. However, it can be difficult to understand the rules used to generate the final classification due to the large number of trees generated from resampling the same dataset. The number of trees, *K*, and predictor variables used at each node, *m*, influence the accuracy of the RF classifier. In this study, different values of *K* (1 to 300) and *m* (1 to 6) were evaluated to optimize these parameters in the final classifier for a total of 1,800 different RF models to predict the risk of SNB. As *K* is increased, the generalization error decreases and converges to a limit (Breiman, 2001). However, the value of *m* (which is constant during forest growth) affects both the correlations between the trees and the strength of the individual trees. Reducing *m* reduces correlation and strength, while increasing *m* increases both. Thus, it is preferable to use a large value for *K* and a small value for *m* to reduce the generalization error and correlation between trees in the forest.

Historically, wheat prices have been low in the United States, which has reduced profit margins for growers (Weisz et al., 2011). Thus, only the most accurate predictor models that guide pre-planting management decisions to minimize unprofitable spray application are likely to be acceptable to risk-averse producers as decision tools. In this regard, the RF model could be a useful pre-planting decision management tool for SNB, as it performed better during internal validation than the other models developed in this study. The model can be used to guide the selection of a specific combination of pre-planting factors that will result in a reduced risk of SNB. Prior to planting the crop, growers can input into the model information on their field location, resistance level of intended cultivar, and the amount of residue in the field. Images of different levels of residue can be provided to growers as references for an estimate of residue levels in their field. Growers can change the combination of their pre-planting factors if the RF model predicts a high SNB risk. In addition, where cultural management practices such as tillage type and crop rotation are difficult to alter in order to reduce the amount of wheat residue, the pre-planting model can provide a quantitative assessment of SNB risk in those situations to facilitate informed decision-making. Although the machine learning models and especially the ANN and RF models developed in this study had a high internal validation accuracy, the models need to be validated with independent data before they can be integrated into a management program for SNB. Such an independent validation of these models would focus on using disease cases collected from growing seasons with a wide range of disease severity levels from locations with diverse cultural practices. Comprehensive economic management decisions for SNB in winter wheat can made by combining prediction models developed in this study with yield-loss models of wheat.

## Author Contributions

LM performed the experiments, collected, and analyzed the data. PO conceived the experiments. LM, PO, and CC designed the experiments. KG provided guidance and assistance on statistical analysis. LM, CC, KG, and PO contributed to writing and approved the final manuscript.

## Conflict of Interest Statement

The authors declare that the research was conducted in the absence of any commercial or financial relationships that could be construed as a potential conflict of interest.
